# Response to the Letter to the Editor on “Psoas Muscle Index as a Predictor of Perioperative Outcomes in Geriatric Patients Undergoing Spine Surgery”

**DOI:** 10.1177/21925682231214658

**Published:** 2023-11-08

**Authors:** Mark N. Pernik, William H. Hicks, Omar S. Akbik, Madelina L. Nguyen, Ivan Luu, Jeffrey I. Traylor, Palvasha R. Deme, Luke J. Dosselman, Kristen Hall, Sarah A. Wingfield, Salah G. Aoun, Carlos A. Bagley

**Affiliations:** 1 Department of Neurological Surgery, 25989 UT Southwestern Medical School, Dallas, TX, USA; 2 Geriatrics Division, Department of Internal Medicine, 25989 UT Southwestern Medical School, Dallas, TX, USA; 3 Department of Orthopedic Surgery, 25989 UT Southwestern Medical School, Dallas, TX, USA

Dear Editors,

The authors thank the writers of the letter for the review and assessment of our article, “Psoas Muscle Index as a Predictor of Perioperative Outcomes in Geriatric Patients Undergoing Spine Surgery.” Overall, we agree that the Psoas Muscle Index requires further validation and study prior to its incorporation in perioperative risk calculations, and we hope our article, and others like it, will contribute to this growing body of literature. In response to the individual comments:

1. In our *Methods* section, we outline the means by which we identified a cut-off for the Psoas Muscle Index (PMI) as follows: “Patients were divided into quartiles based on normalized psoas area measurements. Patients in the fourth quartile (Q4) were compared to patients in quartiles 1 through 3 (Q1-3).” Other studies have utilized tertiles^1^ or treated the PMI as a continuous variable.^2^ As noted in the study, there is a lack of consensus guidelines regarding cutoff values for assessing sarcopenia via the psoas muscle,^3^ and as such, our study sought to contribute to the research surrounding this topic.
2. A limitation highlighted in this study, and many other studies using the psoas muscle, or PMI, as a surrogate for sarcopenia, is the lack of established cut-off values and normalization methods for these studies.^3^ As you pointed out, some studies have defined the PMI by total psoas cross-sectional area to height,^2^ while most have utilized the method we employed, which normalized to the cross-sectional area of the third lumbar vertebrae.^4,5^ We agree that rigorous validation of either method will be important before either method is reliably used in perioperative risk stratification assessments.
3. We agree that standardized imaging protocols should be used for the creation and validation of perioperative risk models fundamentally tied to radiographic data. When measuring PMI on MRI, we used axial T2-weighted sequences, while on CT we used plain axial cuts as described in our methods. Several studies, including the referenced article by Hirase et al 2021, have utilized both CT and/or MRI measurements to calculate the psoas muscle cross-sectional area.^1,2^ As numerical measurements of cross-sectional area are computationally standardized, and PMI does not rely on interpretations of tissue density, composition, or other interpretive data, the authors feel that the imaging modality should have minimal impact on conclusions drawn from each.
4. Indeed, there is a degree of heterogeneity present in the indications for procedure, underlying pathophysiology, and ultimate procedures performed. However, all patients were high-risk geriatric patients (>65 years old) undergoing thoracolumbar surgery. As highlighted in the study, there was no significant difference found in the number of levels instrumented, pelvic fixation, duration of anesthesia, estimated blood loss, and intraoperative blood products needed, indicating a degree of similarity present between the operative groups and the operations performed. A study limited to a population who underwent a single type of operative procedure would increase homogeneity at the expense of generalizability.
